# Rituximab-Induced Immune Dysregulation Leading to Organizing Pneumonia, Bronchiectasis, and Pulmonary Fibrosis

**DOI:** 10.7759/cureus.34798

**Published:** 2023-02-09

**Authors:** Mohammed Ayyad, Jehad Azar, Maram Albandak, Haneen Sharabati, Hamza Salim, Yasmin Jaber, Mohammed Al-Tawil

**Affiliations:** 1 Internal Medicine, Al-Quds University, Jerusalem, PSE; 2 Respiratory Institute, Cleveland Clinic, Cleveland, USA; 3 Department of Medicine, An-Najah National University, Nablus, PSE; 4 Internal Medicine B, Hadassah Ein Kerem Hospital, Jerusalem, ISR

**Keywords:** hypogammaglobulinemia, immunosuppression therapy, granulomatosis with polyangiitis, pulmonary fibrosis, humoral immunity, bronchiectasis, rituximab

## Abstract

We present a case of rituximab-induced organizing pneumonia (OP) along with bronchiectasis and pulmonary fibrosis, in a patient with a history of granulomatosis with polyangiitis (GPA), on long-term maintenance therapy with rituximab. T-cell dysregulation and B-cell depletion associated with the chronic use of rituximab often lead to a profound immunosuppressed state with hypogammaglobulinemia and unbalanced T-cell response. This acquired immunodeficient state with severe immune dysregulation predisposed this patient to recurrent pulmonary infection and ultimately led to bronchiectasis and pulmonary fibrosis.

## Introduction

Rituximab is a monoclonal antibody targeting the antigen CD20 and causing the depletion of CD20+ B-cells [1]. B-cell dysfunction leads to hypogammaglobulinemia, which in turn increases the susceptibility to severe pulmonary infections. Rituximab is also associated with the development of acute fibrinous organizing pneumonia (OP) [2]. Rituximab-induced OP is very rare and has only been described in a few case reports [2-5].

Moreover, rituximab is known to cause drug-induced pneumonitis with fibrosis, as well as hypersensitivity pneumonitis. In this report, we present a case of rituximab-induced hypogammaglobulinemia with subsequent repeated infections, resulting in concurrent bronchiectasis and pulmonary fibrosis.

## Case presentation

We present the case of a 60-year-old male with a history of granulomatosis with polyangiitis (GPA). It manifested as recurrent sinusitis and was associated with end-stage renal disease requiring hemodialysis, despite prior adequate treatment with pulse steroid therapy and rituximab. Consequently, the patient underwent renal transplantation, with subsequent graft dysfunction. Since his diagnosis, the patient had also suffered from recurrent episodes of pneumonia responsive to antibiotics; the patient had continued to undergo steroid therapy as part of his immunosuppressive regimen.

**Figure 1:**
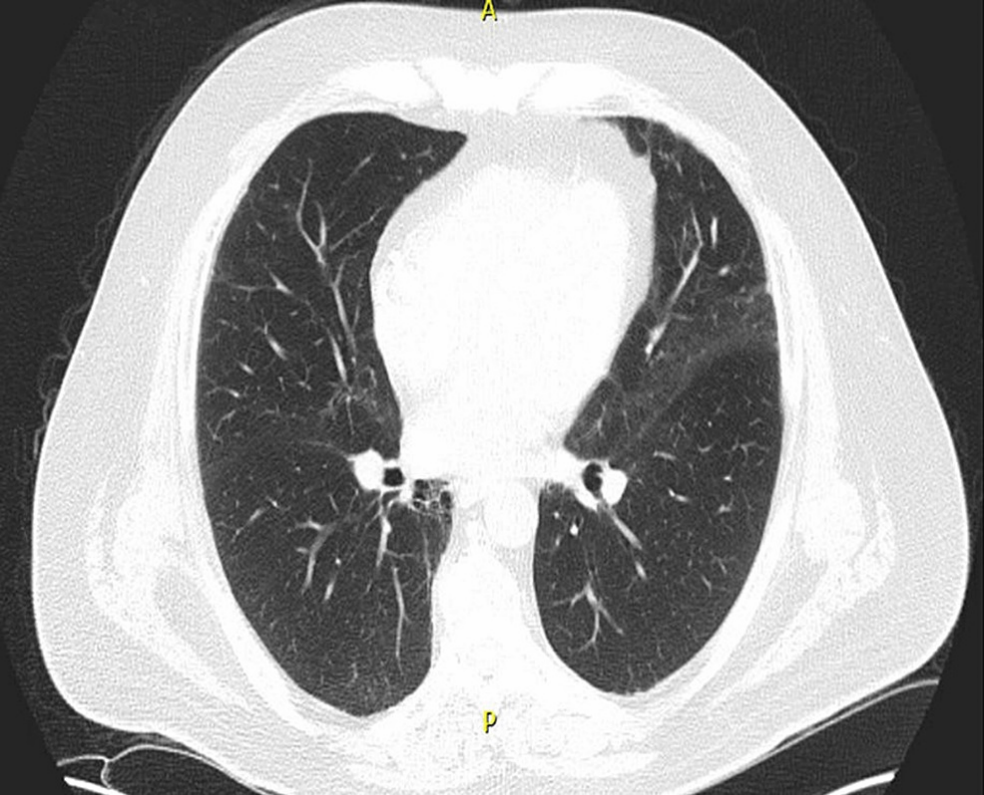
High-resolution CT findings at the time of GPA diagnosis The image shows no signs of interstitial lung disease, bronchiectasis, or consolidation CT: computed tomography; GPA: granulomatosis with polyangiitis

**Figure 2:**
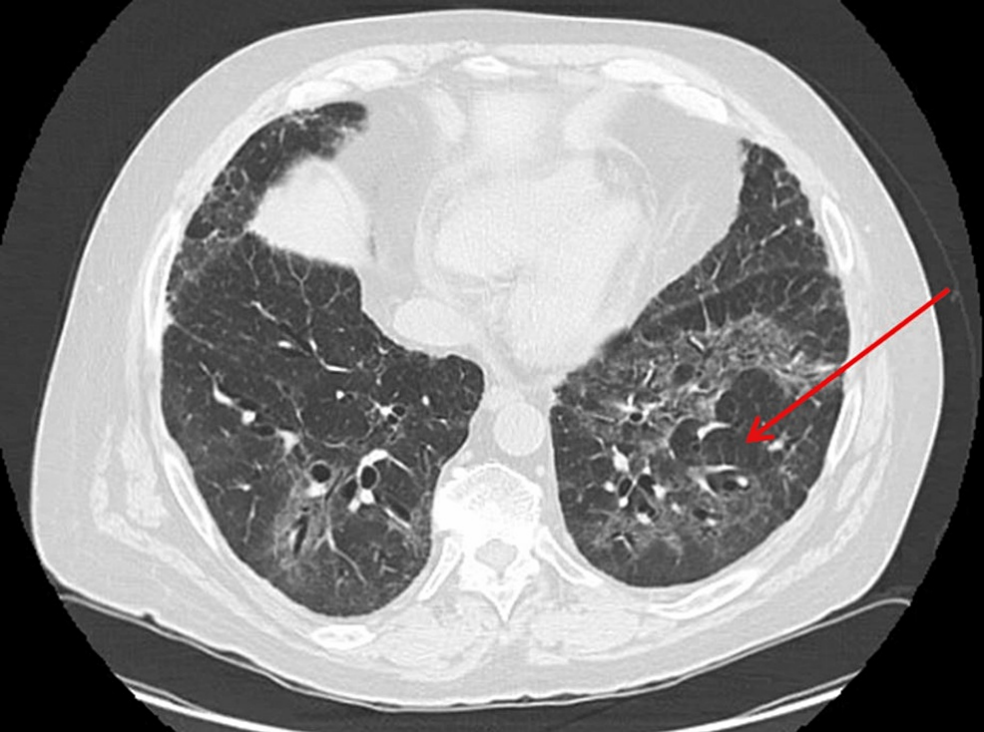
High-resolution CT findings two years after GPA diagnosis High-resolution CT of the chest two years after GPA diagnosis, on rituximab, shows bilateral ground-glass opacities and broncho-centric consolidation, with reversed halo sign (atoll sign) in the left lower lobe (red arrow). It also shows traction bronchiectasis and peripheral reticulation CT: computed tomography; GPA: granulomatosis with polyangiitis

**Figure 3:**
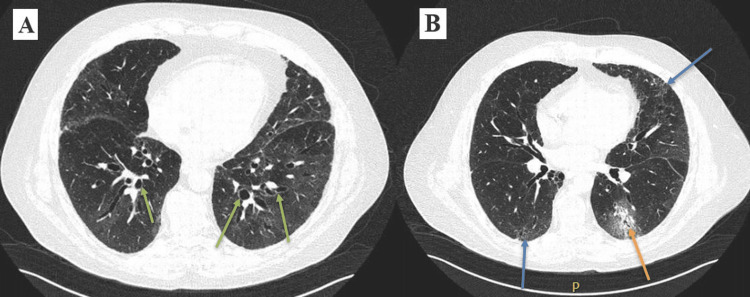
High-resolution CT findings 30 months after GPA diagnosis High-resolution CT chest 30 months after GPA diagnosis, on rituximab, shows bilateral peripheral reticulation (blue arrows), patchy areas of ground-glass opacities and broncho-centric consolidation in the left lower lobe (orange arrow), and traction bronchiectasis associated mild architectural distortion/volume loss (green arrows) CT: computed tomography; GPA: granulomatosis with polyangiitis

Baseline high-resolution CT (HRCT) at the time of GPA diagnosis had revealed no signs of bronchiectasis, consolidation, or interstitial lung disease (Figure 1). However, HRCTs done over the subsequent two years had shown evidence of progressive bronchiectasis, migrating peripheral and broncho-centric consolidations, as well as bilateral fibrotic changes in the form of reticulation, traction bronchiectasis, architectural distortion, and volume loss (Figures 2, 3A, 3B), which are findings atypical to pulmonary GPA.

Based on the patient's clinical and radiological picture, the IgG level test was ordered and was found to be reduced at 530 mg/dL (normal range: 700-1,600 mg/dL). Bronchoalveolar lavage was performed and showed neutrophilic predominance with negative viral, bacterial, and fungal cultures. Pulmonary function tests showed restrictive disease with a moderately reduced diffusing capacity for carbon monoxide (DLCO).

Based on these findings, we suspected that the patient’s immunosuppression was responsible for his recurrent pulmonary infections, bronchiectasis, and fibrosis. Furthermore, the broncho-centric and migratory distribution of the pulmonary consolidation, in addition to the reversed halo sign on HRCT, raised concerns for OP secondary to rituximab therapy and/or repeated chest infections. Due to the lack of clinical improvement after multiple courses of broad-spectrum antibiotics, and the fact that his underlying GPA was deemed to be in a state of remission, the decision was made to resume rituximab therapy at a lower dose, bridged with IVIG supplementation and steroid therapy. This resulted in immunomodulation with B-cell repletion, normalization of immunoglobulin, and T-cell functional recovery. These interventions broke the vicious cycle of immune dysfunction that had led to repeated infection with ultimate bronchiectasis and fibrosis. On follow-up, HRCT was done and showed resolution of OP as well as marked clinical improvement. GPA continued to be in remission and both bronchiectasis and pulmonary fibrosis were stable with no signs of progression. Additionally, it was recommended to check his IVIG levels regularly, with supplementation as required.

## Discussion

Cryptogenic OP is diagnosed by both radiological and clinical presentation. OP, on the other hand, is the histopathological diagnosis [6]. OP can be secondary when associated with conditions known to induce OP, or cryptogenic when the cause is unknown. Secondary OP is associated with viral and bacterial respiratory infections, malignancy, organ transplantation, autoimmune disease, drugs, radiation, and environmental exposure [6]. The classic presentation of OP is a nonproductive cough, malaise, fever, dyspnea, and weight loss, with a preceding mild flu-like illness. This presentation often delays the diagnosis and even makes it difficult to differentiate between secondary infection, preceding infection, and cryptogenic-type OP. In most cases, patients typically receive various courses of antimicrobial agents without clinical improvement, which should indicate the diagnosis of OP.

The pathogenesis of OP is poorly understood [7]. However, T-cell dysregulation appears to be substantially related to OP [8]. It is possible that chronic B-cell depletion induced by rituximab causes an unbalanced T-cell response, which triggers the emergence of OP, especially if the patient is taking other immunomodulating agents [9]. Bronchoalveolar lavage is done to exclude other etiologies, mainly infection, which usually shows differential lymphocytosis. The classic radiological presentation of OP is extensive airspace disease in the form of patchy lower lung zone-predominant consolidation and ground-glass opacities with peri-broncho-vascular and/or subpleural distribution. These are typically bilateral and peripheral and are often migratory, as seen in our case. The atoll sign, which is also known as the reversed halo sign, often seen in cases of OP, is characterized by a central ground-glass opacity surrounded by dense airspace consolidation in the shape of a crescent or a ring. Atoll sign is nonspecific to OP and has been reported in association with a wide range of pulmonary diseases, including invasive pulmonary fungal infections, tuberculosis, community-acquired pneumonia, and GPA [10]. Surgical lung biopsy is the gold standard for diagnosis; however, transbronchial biopsy often shows the diagnostic histopathological findings of excessive fibrotic tissue deposition within alveolar sacs extending into alveolar ducts and bronchioles, as well as intraluminal granulation tissue deposition known as Masson bodies [11]. 

Rituximab-induced OP is rare but has gained interest and recognition recently among physicians given the emerging cases. To the best of our knowledge, there are only a few reported cases of rituximab-induced OP in the literature [2-5]. All of them were treated by withholding rituximab and corticosteroid courses, with subsequent clinical recovery.

Bronchiectasis, on the other hand, is a progressive lung disease characterized by irreversible destruction of the airway due to a combination of immunological dysfunction and recurrent bacterial infection. This leads to progressive bronchiolar dilatation, ciliary dyskinesia, and bacterial colonization and invasion [12]. The original cycle theory of recurrent infections disrupting the integrity of the airway has formed the basis for an emerging version known as the "vicious vortex". This theory proposes that airway inflammation may be due to a dysregulated cytokine network [13,14]. Similarly, rituximab’s side effects are related to its ability to deplete B-cells causing hypogammaglobulinemia with a subsequent susceptibility to infection, as well as immune dysregulation, triggering cytokine imbalance [15]. In our case, we believe that rituximab caused bronchiectasis by the synergistic effect of two mechanisms: immunomodulation and recurrent bacterial infections.

To our knowledge, this sequela of rituximab involving the concurrent development of OP, bronchiectasis, and fibrosis has never been reported before. Moreover, pulmonary fibrosis has been rarely reported as a consequence of long-term rituximab therapy [16]. We suspect that rituximab-induced fibrosis in our patient most likely relates to recurrent infections with a vicious cycle of lung injury and healing with fibrotic tissue deposition.

## Conclusions

Rituximab is a B-cell-depleting agent, and it has detrimental effects on the humoral immune system leading to B-cell depletion with hypogammaglobulinemia and T-cell qualitative dysfunction, with subsequent immune dysregulation. Impaired respiratory mucosal immunity triggers a cascade of recurrent severe pulmonary infections resulting in bronchiectasis and progressive pulmonary fibrosis. Based on our literature review, rituximab is rarely associated with OP. A decision to halt the use of rituximab might be difficult due to the high rates of relapse of the primary illness. Consequently, treating rituximab-associated complications while continuing its administration might be the only feasible approach, with regular evaluation of immunoglobulin levels and supplementation. Steroids or other steroid-sparing agents are used to treat rituximab-induced OP. Airway clearance with bronchopulmonary hygiene, bronchodilation, mucolytics, eradication of pseudomonas colonization, and systemic antibiotics in case of exacerbation are the main treatment modalities for bronchiectasis. Anti-fibrotics should be prescribed if the criteria for progressive pulmonary fibrosis are met.
